# Gradient Artefact Correction and Evaluation of the EEG Recorded Simultaneously with fMRI Data Using Optimised Moving-Average

**DOI:** 10.1155/2016/9614323

**Published:** 2016-06-28

**Authors:** José L. Ferreira, Yan Wu, René M. H. Besseling, Rolf Lamerichs, Ronald M. Aarts

**Affiliations:** ^1^Department of Electrical Engineering, Eindhoven University of Technology, P.O. Box 513, 5600 MB Eindhoven, Netherlands; ^2^Philips Research Laboratories Eindhoven, Professor Holstlaan 4, 5656 AE Eindhoven, Netherlands

## Abstract

Over the past years, coregistered EEG-fMRI has emerged as a powerful tool for neurocognitive research and correlated studies, mainly because of the possibility of integrating the high temporal resolution of the EEG with the high spatial resolution of fMRI. However, additional work remains to be done in order to improve the quality of the EEG signal recorded simultaneously with fMRI data, in particular regarding the occurrence of the gradient artefact. We devised and presented in this paper a novel approach for gradient artefact correction based upon optimised moving-average filtering (OMA). OMA makes use of the iterative application of a moving-average filter, which allows estimation and cancellation of the gradient artefact by integration. Additionally, OMA is capable of performing the attenuation of the periodic artefact activity without accurate information about MRI triggers. By using our proposed approach, it is possible to achieve a better balance than the slice-average subtraction as performed by the established AAS method, regarding EEG signal preservation together with effective suppression of the gradient artefact. Since the stochastic nature of the EEG signal complicates the assessment of EEG preservation after application of the gradient artefact correction, we also propose a simple and effective method to account for it.

## 1. Introduction

Integration of functional magnetic resonance imaging (fMRI) with electroencephalography (EEG) has offered the possibility of understanding new insights into neuroscientific studies because of the higher temporal and spatial measurements of brain activity when compared with the use of each technique separately. Rather than only an additional tool, coregistered EEG-fMRI has been shown to be a promising and powerful technique for the mapping of brain activity and has drawn the attention of several researchers and clinicians in recent years [1–7]. Meanwhile, consolidation of simultaneous EEG-fMRI and enlargement of its range of applications still depend on enhancing the quality of the EEG signal acquired simultaneously with the fMRI data.

The MR scanner constitutes a quite hostile environment for EEG because of the voltages induced by the magnetic fields used for acquisition of fMRI data. Such voltages correspond to three different types of artefact and may corrupt and distort the EEG signal, measured by the scalp electrodes. The first type of artefact is the* movement artefact* associated with motion of the subject head, electrodes, and wires into the static magnetic field (*B*_0_) of the MR scanner, which introduces temporary voltage fluctuations in the measured scalp potential [8, 9]. A second type of artefact is the* pulse* or* ballistocardiogram artefact*, provoked by the pulsatile movement of the blood in scalp arteries within *B*_0_ [10–13]. Finally, the* gradient* or* imaging acquisition artefact* is the voltage induced in the measured scalp potential by the application of rapidly varying magnetic field gradients for spatial encoding of the MR signal and radiofrequency pulses (RF) for spin excitation [14–16]. The occurrence of the movement artefact within a scenario of abrupt head motions as well as the pulse artefact is out of the scope of this work, and further details about their characteristics and methods to suppress them can be found in the abovementioned references.

Regarding the gradient artefact, its amplitudes can be several orders (up to 10^4^ *μ*V) higher than the neuronal EEG signal. The gradient fields and RF pulses used in the MR pulse sequences induce a characteristic and repetitive artefact waveform in the electrical potential picked up by the scalp EEG electrodes (scalp potential), which is approximately the differential waveform of the corresponding gradient pulse [17]. The onset of the artefact waveform corresponds to the occurrence of a RF pulse in the MR sequence, such that the time in-between consecutive RF pulses (termed repetition time, TR) matches the period of the artefact waveform. Typical values of TR range from hundreds of milliseconds to several seconds. The stack of repetitive individual MR slices within a single TR occurs in the recorded scalp potential as signal peaks, and the time corresponding to the acquisition of one slice or slice-time (TR-slice) lies in the range of 50–150 ms. In the frequency-domain, the repetitive feature of the gradient artefact can be observed as discrete harmonic artefact frequency intervals or “frequency bins.” The fundamental of each respective frequency bin corresponds to multiples of the inverse of the slice repetition time (1/TR-slice). For periodic or interleaved fMRI acquisition, in which delays are left between MR volumes, harmonics in the frequency range of 1/TR appear convolved with the frequency bins associated with the slice repetition frequency, 1/TR-slice [15–19].

In the literature, a number of solutions have been proposed to attenuate the effects of the gradient artefact at the source. For instance, it is possible to reduce its magnitude by laying out and immobilising the EEG leads, twisting the leads or modifying the lead paths, using a bipolar electrode configuration, and using a head vacuum cushion [16, 20]. The use of interleaved fMRI acquisition approaches has been shown to be suitable for certain forms of brain activity, such as slowly varying rhythms and evoked responses. However, they are generally less flexible and experimentally efficient than continuous measurements [9, 21]. According to Mullinger et al. [22], the amplitudes of the gradient artefact can also be attenuated by adjusting the subject position within the fMRI scanner. Chowdhury et al. [23] have proposed the use of an EEG cap that incorporates electrodes embedded in an external layer and can record the gradient artefact separately from the EEG signal. Thus, subtraction between the signals recorded by internal and corresponding external electrodes allows the attenuation of the artefact. Although these solutions permit achieving a considerable attenuation of the gradient artefact, its effective suppression and satisfactory EEG correction must be performed by using dedicated postprocessing signal approaches.

The average artefact subtraction (AAS) methodology [14] is the most established postprocessing technique for gradient artefact suppression. Such an approach makes use of the assumption of periodic and stationary nature of the artefact to calculate an average template from occurrences of the artefact waveform, which is then subtracted from the scalp potential. It also assumes the artefact and the EEG signal are not correlated, so that the subtraction of the averaged template permits an estimation of the corrected EEG [24]. The performance of the AAS method highly depends on the reproducibility of the artefact waveform from epoch to epoch, which can be facilitated by utilising a setup that yields more accurate sampling of the gradient artefact waveform over time. Anami et al. [17] and Mandelkow et al. [25] have demonstrated that the use of synchronisation between the fMRI clock and the EEG sampling frequency allows more precise sampling of the artefact and construction of a more accurate artefact template, in consequence. Thus, a cleaner EEG can be obtained after application of AAS in the recorded scalp potential. The performance of AAS has also high dependency on changes in the subject position. Head motions of the subject provoke alterations in the morphology of the artefact waveform over the artefact period, in such a way that the average artefact template cannot characterise individual occurrences of the artefact waveform. To address this problem, Allen et al. [14] and Becker et al. [21] proposed the use of a sliding average window implementation whereby the artefact template may be individually calculated for a particular occurrence of the artefact waveform. However, the correct choice of the number of averaging epochs poses difficulties to implementation of this approach, since few windows can result in removal of the neuronal EEG, whereas the use of many windows can lead to remaining residual artefacts after AAS. Hence, to effectively suppress the gradient artefact with a satisfactory preservation of the neuronal EEG, additional approaches like low-pass filtering with a cut-off frequency around 50–80 Hz and adaptive noise cancelling must be employed to attenuate residual artefacts [14, 15, 20, 21, 26–29].

Some variants of AAS have been devised in attempt to improve the accuracy of template calculation by using principal component analysis [26], independent component analysis [30], and spatial filtering [31]. To correct the jitter between EEG sampling frequency and fMRI clock, more precise computing of the timing error has been addressed by Negishi et al. [32], Gonçalves et al. [33], and Huang et al. [27]. Nevertheless, estimation of an optimal artefact template is still the object of study. In addition, the study of ultra-high-frequency neuronal activity as currently performed [34] requires the use of interleaved approaches as well as customised fMRI sequences that are generally not available to all investigators. Thus, further improvements of AAS and development of novel correction methods are still required and highly desirable to enhance the quality of the EEG signal, mainly regarding EEG signals with low amplitude and with frequency activity in the gamma band (30–100 Hz) and high-frequencies oscillations between 100 and 500 Hz [20, 34].

Because of the risk of simultaneous removal of neuronal EEG activity during application of the gradient artefact correction approach, assessment of the preservation of the EEG signal should be carried out together with the efficacy of the artefact suppression. This, however, has seldom been made systematically or in a consistent way [34–37]. In many EEG-fMRI studies, a single algorithm is chosen without proper justification, and often the quality of gradient artefact correction and EEG preservation is assessed by visual inspection only. The classical (gold standard) way of analyzing EEG signals relies on visual judgement and recognition of sometimes very subtle or short duration phenomena such as spike-wave patterns in epilepsy studies or K-complexes in sleep research. Nonetheless, those patterns may easily be distorted or obscured after application of the artefact correction approach. A difficulty that arises with regard to the analysis of spontaneous EEG excerpts is the stochastic and nonstationary nature of the neuronal EEG. On the other hand, identification of single events in the corrected EEG is not suitable for a scenario in which the signal of interest is the spontaneous EEG and, thus, larger EEG excerpts over time should be analyzed. In addition, the lack of knowledge of the true EEG signal makes it difficult to compare the power spectra of artefact-corrected EEG excerpts with the spectra of the EEG recorded inside or outside the scanner. Thereby, a more systematic approach to assess and compare the performance of the gradient artefact correction methods is advised in some applications, rather than only relying on the analysis of single events or the quantification of EEG power in certain spectral bands. Moreover, to date, generalisation of the correction results for different types of EEG data has been poorly made as well [16, 35–37].

This paper presents a novel methodology for gradient artefact correction based upon optimised moving-averaging (OMA) filtering [38]. OMA filtering constitutes a modality of iterative filtering decomposition [39, 40] and has been exploited in a research project that our group has undertaken to investigate characteristics and features of the gradient artefact that might be used to attenuate, correct, and improve the quality of the corrected EEG signal [38, 41–43]. Optimised moving-average makes use of forward-backward application of a moving-average (MA) filter as an integration procedure to suppress the artefact and estimate partial components of the corrected EEG at the same time. Recursive application of such a procedure allows estimation of the corrected EEG as a sum of the calculated partial components. Rather than estimation of an average template, as performed by the AAS implementation, the artefact is calculated not for epochs, but sample-by-sample, as described in Section 2. To assess the degree of EEG preservation, we have devised a novel and simple evaluation approach that allows accounting for the stochastic nature of the neuronal EEG and was used to perform a comparative analysis of OMA with AAS. Comparison between the performances of OMA and AAS reveals that our method can provide an improved balance for the suppression of the artefact together with a satisfactory preservation of the neuronal EEG signal, as shown in Section 4. In parallel, the use of low-pass filtering or another correction approach to suppress residual artefacts after application of OMA can be avoided, and thus our method potentially better preserves highly relevant high-frequency EEG features. Furthermore, the results indicate that our approach is capable of satisfactorily correcting the EEG data even within a scenario of misalignment between EEG sampling interval and the MR slice-time and without accurate information about MRI triggers. Analysis of the application of the gradient artefact correction in EEG data sets recorded by using MR scanners from two different vendors is also provided in Section 4.

## 2. Methods

### 2.1. EEG and fMRI Data

Our devised methodology was tested in two types of EEG data simultaneously acquired with fMRI data. The EEG data sets were kindly provided by Brain Products GmbH, Gilching, Germany, which gave consent for their publication. Data acquisition was conducted in accordance with the Declaration of Helsinki, approved by the responsible Ethics Committee, and the subjects gave their informed written consent before participating in the study. One of the EEG data sets was recorded within a Philips scanner (hereafter referred to as Philips data), whereas the other EEG data set was recorded within a GE scanner (hereafter referred to as GE data). The EEG data in both the Philips and GE scanner were recorded in two volunteers by using an MRI-compatible 64-channel EEG system (BrainAmp MR, Brain Products GmbH, Gilching, Germany). An MR-compatible EEG cap (BrainCap MR, EASYCAP GmbH, Herrsching, Germany) containing sintered Ag/AgCl electrodes was used to pick up the scalp potential. The EEG cap was arranged in accordance with the standard 10-5 electrodes' positioning, with the FCz position used as reference and the ground electrode located at the AFz position. The impedance of all electrodes was set below 30 kΩ, and one additional electrode was placed on the subject back to record the ECG signal. The EEG amplifiers were positioned inside the scanner bore near the middle axis and connected via fiber optic to a PC interface located outside the scanner room. SyncBox (Brain Products GmbH, Gilching, Germany) was used to synchronise the internal sampling clock of the EEG amplifier and the MRI scanner 10 MHz master clock. The signal acquisition was performed using a sampling rate at 5000 Hz and measurement resolution at 0.5 *μ*V. Hardware-filtering in the frequency band between 0.016 Hz and 250 Hz was applied before data digitalisation in order to prevent saturation and reduce the gradient artefact amplitude. During acquisition, the volunteers were instructed to perform a simple opening/closing eyes manoeuvre at regular time intervals. Regarding acquisition of fMRI data, the following setups were used.

*(A) Philips Data.* Acquisition of the Philips data set was carried out using a 3 T Achieva Scanner (Philips, Eindhoven, The Netherlands). One volunteer was scanned using a functional echo-planar imaging (EPI) sequence with 40 transversal slices and volume repetition time (TR) equal to 2000 ms. The fMRI clock and the EEG sampling frequency have been synchronised, so that TR was set as a multiple of the EEG sampling interval. fMRI data acquisition was continuously performed, and TR was adjusted as a multiple of the slice-time (TR-slice). Hence, TR-slice was equal to 2000 ms/40 slices = 50 ms. Acquisition of the Philips data was approximately performed during 4 minutes. 

*(B) GE Data.* Acquisition of the GE data set was carried out using a 3 T Discovery MR750 Scanner (GE, Waukesha, USA). A second volunteer was scanned using an EPI sequence with 28 transversal slices and volume repetition time (TR) equal to 2000 ms. fMRI data acquisition was continuously performed, and the fMRI clock and the EEG sampling frequency have been synchronised for a period equal to 500 ms, corresponding to seven times of TR-slice. Thereby, although TR was approximately adjusted as a multiple of the slice-time (TR-slice), TR-slice was not aligned and did not match a multiple of the EEG sampling interval. Acquisition of the GE data was approximately performed during 10 minutes.

### 2.2. Proposed Methodology for Gradient Artefact Correction

Our proposed methodology for gradient artefact correction was implemented in two steps: (i)* peak detection and TR-slice estimation* and (ii)* optimised moving-average filtering*. The recorded scalp potential, *s*_*n*_, in one specific EEG channel was mathematically modelled as a linear superposition of the neuronal EEG, *e*_true,*n*_, and the induced voltage associated with the gradient artefact interference, *g*_artf,*n*_:

(1) $$\begin{array}{c} s_{n}=e_{true,n}+g_{artf,n}, \end{array}$$

where *n* is the time sample.

### 2.3. Peak Detection and TR-Slice Estimation

An initial detection of the peaks corresponding to the onset of the MR slices observed in the recorded scalp potential must be performed according to our proposed methodology. Such detection permits estimation of the slice-time (TR-slice) according to the time basis of the EEG sampling system, which is utilised during implementation of the optimised moving-average filtering. Within a scenario of alignment between the MR slice-time and the EEG system sampling interval, the estimated TR-slice precisely corresponds to a multiple of the EEG sampling interval [25, 28, 29]. However, when there is misalignment between the MR slice-time and the EEG sampling interval, the value estimated for TR-slice may not match a multiple of the sampling interval and, thereby, vary. This variation can be accounted for by application of optimised moving-average filtering, as described in Section 2.4.

### 2.4. Optimised Moving-Average Filtering (OMA)

**Figure 1:**
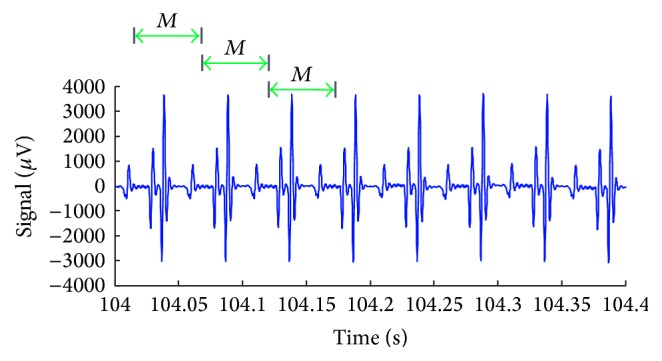
Scalp potential excerpt recorded during continuous acquisition of fMRI data. Assuming that the artefact waveform is stationary, any moving-average window of length *M* (= TR-slice) along the signal contains the artefact waveform corresponding to one slice, but with onset other than of those samples localised in the signal peaks.

Figure 1 shows an excerpt of the scalp potential picked up from the Philips data, in which TR is a multiple of TR-slice. The time measured between two consecutive peaks in the signal matches the slice-time or TR-slice. Assuming that the artefact waveform is stationary, any moving-average window *M* with length equal to TR-slice along the signal contains the artefact waveform period, but with a different onset of those samples localised in the signal peaks. Thereby, assuming that the gradient artefact waveform is stationary and has zero mean, integration of (1) over the period *M* results in cancellation of the artefact waveform. Also assuming that the terms of (1) are uncorrelated, the resulting value of the integral along the scalp potential, **s**, corresponds to a mean estimate of the neuronal EEG, $\bar{{\hat{e}}_{n}}$. This integral can be described as a moving-average filter with order *M*:

(2) 1M∑k=0M−1sn−k1M∑k=0M−1etrue,n−k+gartf,n−k

(3) 1M∑k=0M−1etrue,n−k+1M∑k=0M−1gartf,n−k=e^n¯.

Because of the phase distortion provoked by the moving-average filter [44–46], the mean value $\bar{{\hat{e}}_{n}}$ is not in phase with the neuronal EEG, *e*_true,*n*−*k*_. In order to make them in phase, the moving-average must be backward applied in (3):

(4) 1M∑k=M−10e^n+k−=1M∑k=M−101M∑k=0M−1sn−kn+k=1M2sn−M+1+2sn−M+2+⋯+M−1sn−1+Msn+M−1sn+1+⋯+2sn+M−2+sn+M−1

(5) =1M2∑k=−M+1M−1M−ksn+k=ecomp,1,n.

Thereby, according to (4) and (5), forward-backward application of the moving-average filter in the recorded scalp potential results in the signal **e**_comp,1_ that is in phase and constitutes a mean approximation of the neuronal EEG [38]:

(6) $$\begin{array}{c} e_{comp,1}\approx {\hat{e}}_{true}. \end{array}$$

Equation (5) acts as a smoothing filter, in such a way that the signal **e**_comp,1_ contains low-frequency activity associated with ${\hat{e}}_{true}$. In turn, the frequency activity associated with the gradient artefact is contained in the signal, **e**_high,1_, resulting from the subtraction of **e**_comp,1_ from** s**:

(7) $$\begin{array}{c} e_{high,1}=s-e_{comp,1}. \end{array}$$

Since high-frequency components associated with ${\hat{e}}_{true}$ remain in **e**_high,1_, it is possible to obtain an estimate of such components by the iterative application of (5) in **e**_high,1_. The second component, *e*_comp,2,*n*_, results from the application of (5) in **e**_high,1_:

(8) $$\begin{array}{c} e_{comp,2,n}=\frac{1}{{\left(M\right)}^{2}}\overset{M-1}{\underset{k=-M+1}{\sum }}\left(\;M-\left|\;k\;\right|\;\right)e_{high,1,n+k}, \end{array}$$

and the signal **e**_high,2_ can be obtained afterwards:

(9) $$\begin{array}{c} e_{high,2}=e_{high,1}-e_{comp,2}. \end{array}$$

This procedure was repeated so forth, for a number *j* of iterations, allowing estimation of the component **e**_comp,*j*_:

(10) $$\begin{array}{c} e_{comp,j,n}=\frac{1}{{\left(M\right)}^{2}}\overset{M-1}{\underset{k=-M+1}{\sum }}\left(\;M-\left|\;k\;\right|\;\right)e_{high,j-1,n+k}. \end{array}$$

This procedure also constitutes a modality of iterative filtering decomposition (IFD) in which (5) is termed double average filter [39, 40]. The convergence of IFD has been demonstrated in Lin et al. [39] and is ensured by the coefficients (masks) of the double average filter having value between 0 and 1.

Finally, the estimate ${\hat{e}}_{true}$ of the corrected EEG can be calculated by the sum of *J* estimated components:

(11) $$\begin{array}{c} {\hat{e}}_{true}=\overset{J}{\underset{j=1}{\sum }}e_{comp,j}. \end{array}$$

**Figure 2:**
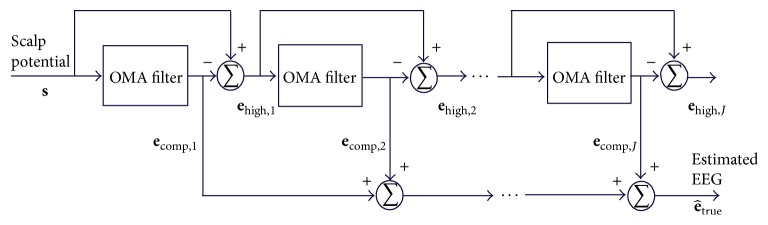
Scheme of the optimised moving-average filtering (OMA): estimation of the corrected EEG (${\hat{e}}_{true}$) is carried out by recursive application of (10) in the scalp potential and successive **e**_high,*j*_, followed by subtraction of the components **e**_comp,*j*_. The blocks labelled as OMA filter represent the forward-backward moving-average filtering, as described in (10).

Implementation of (11) can be visualised in the scheme of Figure 2.

Application of the *z*-transform in the OMA filter, *H*_OMA_, described in (10), permits finding its transfer function:

(12) $$\begin{array}{c} H_{OMA}\left(\;z\;\right)=\frac{1}{{\left(M\right)}^{2}}\frac{\left(1-z^{-M}\right)\left(1-z^{M}\right)}{\left(1-z^{-1}\right)\left(1-z\right)}. \end{array}$$

**Figure 3:**
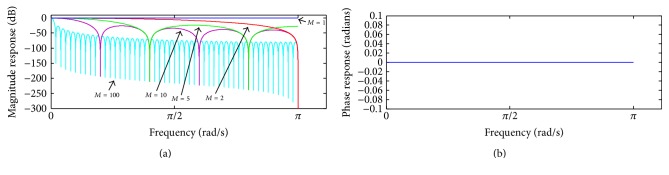
Frequency response of the OMA filter for some values of *M*: (a) magnitude response; (b) phase response. The phase response is the same for any values of *M*.

Making use of (12), the frequency response of *H*_OMA_ can be calculated by setting *z* = *e* ^*jω*^.  Figure 3 shows such a frequency response for some values of *M*.

The magnitude response reveals the presence of spaced zeros at the frequency 2*π*/*M*. For a hypothetical value *M* = 1, the OMA filter represents an all-pass band filter, as expected for a moving-average filter. The phase response confirms the zero-phase characteristic of the OMA filter. From (7), we can find the transfer function between **e**_high,1_ and **s**:

(13) H11−1M21−z−M1−zM1−z−11−z=1−HOMAz.

Hence, **e**_high,*J*_ corresponds to

(14) $$\begin{array}{c} E_{high,J}\left(\;z\;\right)={\left[\;1-H_{OMA}\left(\;z\;\right)\;\right]}^{J}S\left(\;z\;\right). \end{array}$$

Therefore, (12), (13), and (14) allow removing *J* cascade components shown in Figure 2 and establishing the transfer function between ${\hat{e}}_{true}$ and **s**:

(15) $$\begin{array}{c} \frac{{\hat{E}}_{true}\left(z\right)}{S\left(z\right)}=1-{\left[\;1-H_{OMA}\left(\;z\;\right)\;\right]}^{J}=H_{C}\left(\;z\;\right). \end{array}$$

**Figure 4 fig4:**
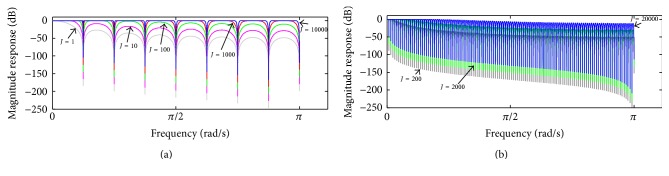
Magnitude response of (15), taking into account (a) *M* = 16, (b) *M* = 250, and some values of *J*.

In Figure 4, the magnitude response of *H*_*C*_(*z*) in (15) is depicted, taking into account *M* = 16 and *M* = 250 and some values of *J*. It can be observed that increasing of *J* is followed by substantial droop reduction (increasing gain) in the filter pass-bands. On the other hand, increasing of *J* is also followed by reduction in the attenuation in the filter stop-bands.

**Figure 5 fig5:**
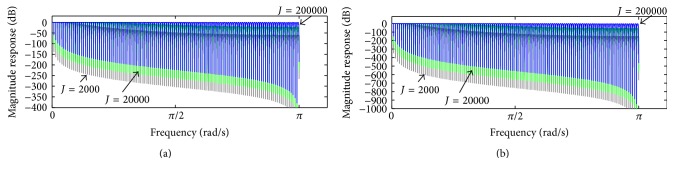
Magnitude response of (16) for (a) *L* = 2 and (b) *L* = 5, taking into account *M* = 250 and some values of *J*.

In order to improve the attenuation in the stop-bands, *H*_*C*_(*z*) might be applied within a cascade implementation as indicated in

(16) $$\begin{array}{c} H_{L}\left(\;z\;\right)={\left[\;H_{C}\left(\;z\;\right)\;\right]}^{L}, \end{array}$$

where *L* is the number of cascades. Thereby, (16) equals (15) when *L* = 1.  Figure 5 depicts the magnitude response of *H*_*L*_(*z*), for *L* = 2 and *L* = 5, taking into account *M* = 250 and some values of *J*. As noticed, (16) can be used to provide reduced droop in the pass-bands together with higher attenuation in the stop-bands according to the values of *J* and *L*. In addition, (15) and (16) possess a zero-phase characteristic as well, similar to that observed in Figure 3(b).

Therefore, (15) and (16) can be used to estimate the artefact-corrected EEG in *z*-domain. On the other hand, in time-domain, the corrected EEG, ${\hat{e}}_{true}$, can be calculated by using either (11) or (17):

(17) $$\begin{array}{c} {\hat{e}}_{true}=s-e_{high,J}. \end{array}$$

As indicated in (10) and (12), the estimate **e**_comp,*j*_ is calculated sample-by-sample, rather than calculation and subtraction of an average artefact template. Thus, the optimised moving-average filtering implementation depicted in Figure 2 permits an individual calculation and subtraction of the artefact for each signal sample rather than epochs averaging, as performed by AAS. Hence, the inherent uncertainty associated with averaged samples and the influence of alterations of the artefact waveform provoked by subject head motions might be minimised by using our proposed approach. To assess the performance of the proposed approach in a scenario of misalignment between the EEG sampling interval and the slice-length, OMA has been applied by using (16), taking into consideration some values of *L*, as shown in Section 4.

## 3. Evaluation of the Gradient Artefact Correction and Comparative Analysis

According to Ritter et al. [35], two measures must be complimentarily used to evaluate the performance of the gradient artefact correction approach: (i) the effectiveness of gradient artefact attenuation and (ii) the degree of preservation of the neuronal EEG after artefact correction.

### 3.1. Assessment of the Artefact Attenuation

To assess the gradient artefact attenuation, the RMS and amplitude of the artefact voltage over time were calculated taking into account the subtraction between** s** and the estimation of the corrected EEG, ${\hat{e}}_{true}$. Also we performed calculation of the spectral power attenuation around the fundamental of the frequency bins associated with the slice repetition frequency (1/TR-slice). To this end, we have estimated and took into consideration a bandwidth of ±1 Hz around the fundamental of each frequency bin. Calculation of the spectral power attenuation was carried out for the frequency bins below 500 Hz. Although a band limiter set at 250 Hz was employed during data acquisition, we would rather evaluate the attenuation in frequency bins up to 500 Hz because of the artefactual energy that may remain above the band limiter edge frequency [47]. Equation (18) was used to compute the spectral power attenuation in decibel:

(18) $$\begin{array}{c} Attenuation=-20\times \log\left(\;\frac{P_{A}}{P_{B}}\;\right)\;dB, \end{array}$$

where *P*_*B*_ and *P*_*A*_ correspond to the spectral power within the harmonic artefact bins, before and after application of the gradient artefact correction, respectively.

### 3.2. Evaluation of the EEG Preservation

**Figure 6 fig6:**
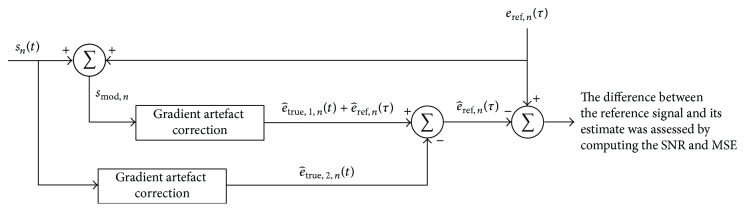
Proposed scheme for measurement of the EEG signal preservation by linear addition of an artefact-free EEG excerpt, *e*_ref,*n*_, in the recorded scalp potential, *s*_*n*_. Both *e*_ref,*n*_ and *s*_*n*_ were picked up from the same EEG channel at different times, *t* and *τ*. The blocks labelled as* gradient artefact correction* match one specific gradient artefact correction approach (OMA or AAS).

The scheme depicted in Figure 6 was used to perform the quantitative evaluation of the EEG preservation.

As indicated in Figure 6, a reference EEG signal, *e*_ref,*n*_, has been linearly added to the measured scalp potential, *s*_*n*_, thus generating the modified signal *s*_mod,*n*_. As *e*_ref,*n*_, we used EEG excerpts recorded inside the MR scanner during nonscan periods. Thereby, the letters *τ* and *t* indicate that the reference EEG excerpt has been recorded at a different time than *s*_*n*_. The gradient artefact correction was then applied to *s*_mod,*n*_ and *s*_*n*_, resulting in the estimates ${\hat{e}}_{true,1,n}$ + ${\hat{e}}_{ref,n}$ and ${\hat{e}}_{true,2,n}$, respectively. Thereby, the subtraction between these estimates allows obtaining an estimate of the reference signal, ${\hat{e}}_{ref,n}$, which was finally compared with *e*_ref,*n*_. Equations (19) and (20) were used to calculate the signal-to-noise ratio (SNR) and the mean squared error (MSE) as complimentary measures of temporal and frequency contents of ${\hat{e}}_{ref,n}$ in comparison with *e*_ref,*n*_:

(19) SNR=coveref,e^refσeref·σe^ref,

(20) MSE=1N∑n=1Neref,n−e^ref,n2.

The SNR calculated by (19) corresponds to a measure of cross-correlation and allows an evaluation and comparison of frequency characteristics between *e*_ref,*n*_ and ${\hat{e}}_{ref,n}$ [46]. Values of SNR closer to unity mean higher similarity between *e*_ref,*n*_ and ${\hat{e}}_{ref,n}$. In turn, smaller values of MSE computed according to (20) indicate higher correspondence between *e*_ref,*n*_ and ${\hat{e}}_{ref,n}$ over time. In addition to being of simple implementation, the evaluation scheme of Figure 6 has the advantage of allowing the assessment of longer EEG excerpts by accounting for the stochastic nature of the neuronal EEG signal and not single events only.

### 3.3. Comparative Analysis

The results obtained by application of OMA were compared with those obtained by subtraction of a mean template, according to the average artefact subtraction (AAS) methodology [14, 21]. Both OMA and AAS were applied to the Philips data and GE data set. For implementation of OMA, we developed and applied our proposed methodology to the EEG recordings in MATLAB (The MathWorks Inc., Natick, USA) environment. In turn, the average artefact subtraction was carried out by utilising the software Brain Vision Analyzer 2 (Version 2.1.0.327; Brain Products GmbH, Gilching, Germany). As benchmark, a sliding moving-average window implementation of 21 epochs was used for construction of the average artefact template. The reason for using 21 epochs was based upon the default settings of the Brain Vision Analyzer. The epoch length to be averaged and construct the artefact template was set at 50 ms (TR-slice) for the Philips data and 500 ms for the GE data. Such values were chosen to match the minimum period in which the fMRI data were synchronised as a multiple of the EEG sampling interval, so that the influence of head motions in longer sliding average windows might be prevented.

After application of OMA and AAS, assessment of artefact attenuation and evaluation of the EEG preservation, as described above, were carried out using MATLAB. All 63 EEG channels were used to perform the analysis of the GE data. For the Philips data, the channel TP8 was excluded from the analysis because the entire recordings were corrupted by artefacts, which made it impossible to pick up a signal excerpt representative of *e*_ref,*n*_.

## 4. Results

**Figure 7:**
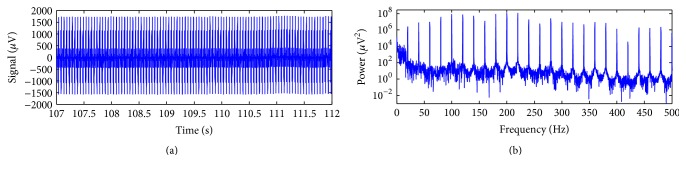
(a) Scalp potential excerpt picked up from the EEG electrode Fz of the Philips data; (b) power spectrum of the signal (a), showing up the harmonic artefact activity associated with the gradient artefact at multiples of 1/TR-slice (TR-slice = 250 samples), equal to 20 Hz for the Philips data.

Figure 7(a) depicts an exemplary scalp potential excerpt, picked up from the Philips data, electrode position Fz. In Figure 7(b), the power spectrum of the signal of Figure 7(a) is shown, and the harmonic activity associated with the gradient artefact can be visualised as spectral peaks at multiples of the fundamental frequency equal to 20 Hz, corresponding to the frequency bins associated with 1/TR-slice.

**Figure 8 fig8:**
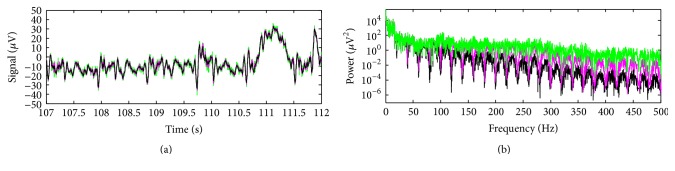
(a) Scalp potential excerpt of Figure 7(a) after application of OMA, taking into account (15), for *J* = 200 (dark trace), *J* = 2000 (pink trace), and *J* = 200000 (green trace); (b) power spectra corresponding to the signals of (a).

By performing the peak detection associated with the gradient artefact in the signal of Figure 7(a), TR-slice was estimated in even lengths of 250 samples, thus confirming the alignment between TR-slice and the EEG sampling interval for the Philips data. Hence, for application of the optimised moving-average (OMA), we set *M* = 250.  Figure 8(a) depicts the artefact-corrected EEG by using (15), for *J* = 200, *J* = 2000, and *J* = 200000. It can be seen in Figure 8(b) that the harmonic activity associated with the gradient artefact has been attenuated for the used values of *J*. Increasing the value of *J* is shown to provoke better preservation of the power activity along the spectrum of the corrected EEG signal, mainly in high-frequencies. Smaller attenuation in the frequency bins associated with 1/TR-slice is also noticed when the value of *J* is increased, which agrees with the comb-filtering response depicted in Figure 4 as well. Therefore, the choice of the value of *J* should be made in such a way as to provide adequate attenuation of the artefact activity in the frequency bins and satisfactory preservation of the EEG signal. However, (15) might not provide enough attenuation of the artefact, and (16) can be used instead, as shown in the next example.

**Figure 9 fig9:**
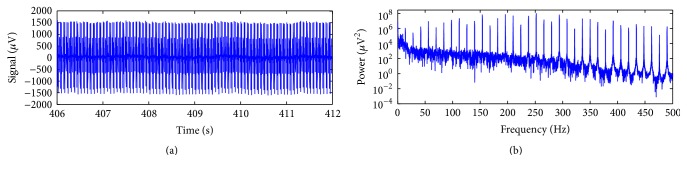
(a) Scalp potential excerpt picked up from the EEG electrode FP1 of the GE data; (b) power spectrum of the signal (a), showing up the harmonic artefact activity associated with the gradient artefact at multiples of 1/TR-slice (TR-slice = 357 ± 1 samples), approximately equal to 14 Hz for the GE data.

An illustrative scalp potential excerpt picked up from the GE data, EEG electrode position Fp1, is depicted in Figure 9(a), and its corresponding power spectrum is shown in Figure 9(b).

**Figure 10 fig10:**
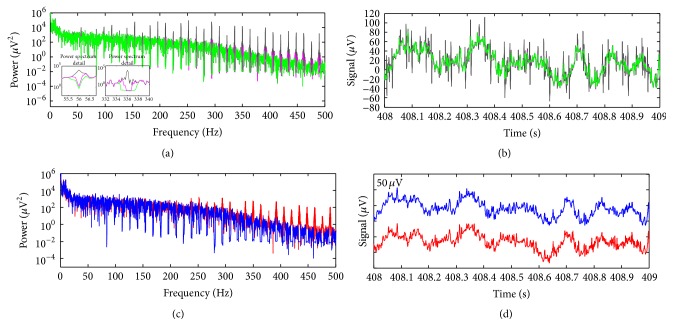
(a) Power spectrum of the scalp potential of Figure 9(a) after application of OMA, taking into account (16) and *J* = 200000, for *L* = 1 (dark trace), *L* = 5 (pink trace), and *L* = 100 (green trace); (b) detail in the time-course corrected EEG by OMA with similar setup and colour code as indicated in (a); (c) power spectrum of the scalp potential of Figure 9(a) taking into account (16) for *L* = 100 (blue trace) and power spectrum of the corrected EEG by AAS (red trace); (d) detail in the time-course corrected EEG by OMA (blue trace) and AAS (red trace).

For these data, the slice-length has not been aligned with the EEG sampling interval, in such a way that the value of TR-slice was estimated at 357 ± 1 samples. To demonstrate the performance of the proposed method to suppress the gradient artefact in this scenario, we used (16), taking into account *M* = 357 and *J* = 200000, for *L* = 1 (see (15)), *L* = 30, and *L* = 100.  Figure 10(a) depicts the corresponding power spectra of the corrected EEG. For *L* = 1, OMA was unable to satisfactorily attenuate the gradient artefact, so that residual spectral power artefact remained in most of artefact frequency bins (dark trace).  Figure 10(b) shows a detail of the time-course corrected EEG signals around 408 s in which the presence of such residuals can also be noticed (thin dark traces). Nevertheless, higher values of *L* allow increased attenuation of the harmonic artefact activity (Figures 10(a) and 10(b)). For *L* = 5 (pink trace), residual spectral power associated with the artefact was substantially attenuated in the bandwidth up to 300 Hz. In turn, when *L* = 100 (green trace), such attenuation was even higher, and artefact harmonic activity in the frequency bins could be strongly reduced in the bandwidth below 500 Hz.

As observed in the spectrum details around 56 and 336 Hz (Figure 10(a)), by increasing *L*, it provokes larger attenuation around the frequency bins. Thereby, (16) can be used to account for the enlargement of the spectral artefact harmonic lines provoked by the alignment error between the EEG sampling interval and TR-slice, thus being able to correct the scalp potential within this scenario. No signal interpolation for correction of the artefact waveform phase has been performed.  Figure 10(c) depicts the power spectra of the corrected EEG after application of OMA (blue trace, *J* = 200000 and *L* = 100) and the mean template subtraction by AAS (red trace) in the signal of Figure 9(a). As can be noticed for the signal corrected by the AAS method, residual power associated with the artefact activity arose in higher-frequencies bins, above around 200 Hz. In Figure 10(d), a detail around 408 s of the time-course of the corrected EEG by OMA and AAS is also depicted. Some small amount of residual artefacts corresponding to the artefact residual power can be noticed in the time-course signal corrected by AAS. Rather, those residuals could be attenuated by using OMA.

Both OMA and AAS play a role of comb-filtering approaches [24, 38] whereby harmonic frequency components associated with the slice repetitive frequency (1/TR-slice) can be attenuated. On the one hand, AAS implementation consists of a coherent detection-based comb-filtering process [24, 48] that is carried out by subtraction of the template with period TR-slice. As such, the AAS method is highly dependent on precise sampling of the scalp potential as well as accurate alignment amongst the averaging epochs to construct the average template. Moreover, small drifts and subject head motions can provoke broadening of the high-frequency artefact spectral lines, in such a way that AAS may fail to attenuate them, and residual artefacts arise in the corrected EEG as a consequence [49]. This helps to explain why AAS is not effective in eliminating the high-frequency artefact activity shown in Figures 10(c) and 10(d), whose attenuation is more affected by imprecise sampling than low-frequency artefact activity as well [25, 29]. On the other hand, OMA performs comb-filtering making use of the filtering implementation described in Section 2.4. By using proper values of *M*, *J*, and *L* in (16), thereby, it allows OMA to effectively account for the attenuation of high-frequency artefact activity, as depicted in Figure 10.

The results presented in Tables 1 [Table tab2] [Table tab3] [Table tab4]–5 for the OMA correction (16) refer to *J* = 200000 and *L* = 1 (Philips data) and *J* = 200000 and *L* = 100 (GE data). As observed in Table 1, the median RMS and amplitude calculated for the artefact voltages estimated by both approaches are quite similar. However, when the power spectra of the corrected signals are compared, the attenuation of the artefact activity in the frequency bins provoked by the AAS and OMA is different (Table 2). Although both approaches are shown to provoke attenuation approximately similar in some artefact frequency bins, OMA provided more attenuation than AAS in higher-frequency bins.

To perform the quantitative evaluation of EEG preservation, we used the scheme depicted in Figure 6 and calculated the median values of SNR (19) and MSE (20) between *e*_ref,*n*_ and ${\hat{e}}_{ref,n}$. The results of such measures are shown in Tables 3, 4, and 5. In Tables 4 and 5, we show the individual results for some exemplary channels, Fp1, F3, Oz, CPz, Fz, FC5, and AF3. In these tables, we also included the values of SNR and MSE, considering application of low-pass (LP) filtering in *e*_ref,*n*_ and ${\hat{e}}_{ref,n}$. The LP filter cut-off frequencies are indicated and were used to assess the EEG preservation in different EEG bandwidths for both OMA and the AAS method. Calculation of the median global values shown in Table 3 also took into account the results considering LP filtering. It can be observed that the overall values of SNR and MSE for the OMA method are better than those for AAS.

It is also noteworthy that low-pass filtering of *e*_ref,*n*_ and ${\hat{e}}_{ref,n}$ substantially increased the SNR and decreased the MSE considering the OMA approach, unlike the AAS method. Therefore, by using OMA, the signals *e*_ref,*n*_ and ${\hat{e}}_{ref,n}$ have become more similar after LP filtering, attesting even better preservation of the neuronal EEG in low-frequencies. We also noticed that the mean subtraction by AAS produced signals *e*_ref,*n*_ and ${\hat{e}}_{ref,n}$ less similar because of the higher influence of small drifts and subject head motions. This led to the small differences of the values of SNR and the MSE for the considered LP cut-off frequencies, as observed in Tables 4 and 5 (AAS).

**Figure 11:**
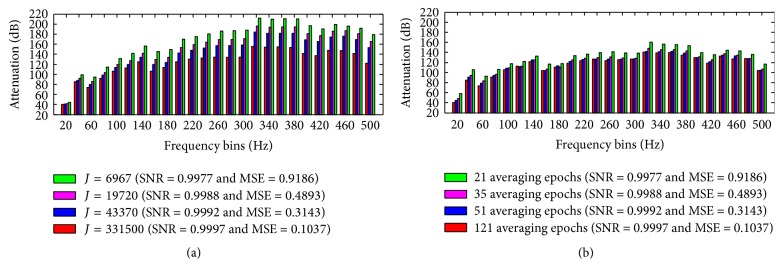
Attenuation in the frequency bins provoked by application of (a) OMA and (b) AAS for an EEG excerpt picked up from the channel CPz (Philips data), taking into account different values of *J* and number of averaging epochs. Although OMA and AAS can provide similar EEG preservation (same value of SNR and MSE), OMA is more effective in attenuating the artefact in the overall frequency bins, mainly in higher-frequencies than 100 Hz.

Figure 11 shows a comparison of the typical attenuation in the frequency bins provoked by OMA (15) and AAS, taking into account different values of *J* and different number of averaging epochs, as well as similar EEG preservation (same SNR and MSE). The results shown in Figure 11 were obtained from an EEG excerpt picked up from the channel CPz of the Philips data. As observed, even though OMA and AAS can provide similar EEG preservation according to the values of *J* and the number of averaging epochs, respectively, OMA provokes larger attenuation than AAS in the overall frequency bins, mainly in frequencies higher than 100 Hz. In addition, the larger the values of *J*, the higher the difference of attenuation by OMA in a certain frequency bin. In turn, the attenuation by AAS is more uniform among different numbers of averaging epochs taking into consideration a certain frequency bin. The attenuation provided by AAS is shown to be slightly higher and less uniform than OMA only for the frequency bins 20 and 40 Hz. Therefore, Figure 11 demonstrates that our proposed comb-filtering approach can be more effective in attenuating the artefact activity and simultaneously in achieving EEG preservation similar to that provided by the AAS method.

## 5. Discussion

As the repetitive gradient artefact waveform recorded in the scalp potential during continuous acquisition of EEG-fMRI is approximately the differential waveform generated within the MR sequence [17], a proper integration over the artefact period might be used to cancel out the artefact, as indicated in Figure 1 and (3). To implement such integration, we have investigated and proposed the forward-backward application of a moving-average filter in the scalp potential, described in (5). When this procedure is recursively carried out, as depicted in the scheme of Figure 2, it permits suppressing the gradient artefact from the scalp potential and obtaining an estimation of the neuronal EEG [38]. Such kind of moving-average procedure is referred to as iterative filtering decomposition, which has been investigated as an alternative implementation for empirical mode decomposition [39, 40]. It also constitutes a comb-filtering approach whereby harmonic signal components can be filtered out (Figures 3, 4, and 5). As observed in Figures 4 and 5, the OMA comb-filtering approach described in (15) and (16) can provide increased gain in the filter pass-bands together with effective attenuation in the stop-bands, in addition to possessing a zero-phase characteristic (Figure 3 [Fig fig4] [Fig fig5] [Fig fig6]). Thus, it is able to effectively suppress the harmonic artefact activity associated with the repetitive artefact waveform that occurs in the slice repetition time (TR-slice) and satisfactorily preserve the neuronal EEG signal at the same time, as observed in Figures 7 [Fig fig8] [Fig fig9] [Fig fig10]–11 and Tables 1 [Table tab2] [Table tab3] [Table tab4]–5.

When compared with subtraction of an average artefact template, according to the AAS method implementation [14, 21], both OMA and AAS show a quite similar RMS and amplitude attenuation associated with the gradient artefact over time (Table 1). Meanwhile, OMA is shown to provoke different attenuation in the artefact frequency bins in comparison with AAS, as can be observed in Table 2 and Figure 11. As a consequence, our proposed method can lead to different preservation of the EEG signal than AAS, as indicated by the SNR and MSE (Tables 3, 4, and 5). In addition, taking into account similar EEG preservation (same SNR and MSE), OMA is shown to be more effective in attenuating the artefact in the overall frequency bins (Figure 11), especially in higher-frequencies (above 100 Hz). Thus, this characteristic can avoid the use of further processing methods such as adaptive noise cancelling (ANC) and low-pass (LP) filtering that may contribute to suppressing high-frequency activity of the neuronal EEG. This is a substantial conceptual advantage compared to AAS, in which postprocessing is essential to remove residual artefacts [14, 25, 26, 28]. Therefore, these results indicate that OMA can lead to a better balance for the trade-off artefact attenuation and EEG preservation, thus outperforming the slice-average subtraction by AAS. Better preservation of the corrected EEG in low-frequencies was also achieved by OMA, as shown in Tables 3, 4, and 5. The SNR closer to unity and the smaller values of the MSE reflect smaller difference between the reference EEG, *e*_ref,*n*_, and its estimate, ${\hat{e}}_{ref,n}$, after application of OMA than AAS. Thus, it confirms an improved preservation and less distortion of the EEG signal by using OMA.

Yet, baseline correction has not been performed before application of OMA, whereas it has been carried out before AAS. This evidences that OMA is more robust to the alterations of the artefact waveform caused by small drifts and movements of the subject head. The inherent uncertainty (standard deviation) associated with averaging epochs provoked by alterations in the artefact waveform because of small drifts as well as subject head motions may lead to an inaccurate estimation of the average artefact template [15]. Contrary to AAS, gradient artefact estimation by our method does not rely on calculation of an average template. Rather, implementation of OMA is individually performed sample-by-sample according to application of the OMA filter indicated in (10), whose uncertainty is influenced by artefact waveform alterations that occur in samples of the scalp potential raging from *s*_*n*−*M*+1_ to *s*_*n*+*M*−1_. In case of AAS, it is affected by artefact waveform alterations that occur in those 2*m* + 1 sliding average windows considered for average template construction, whose samples range from *s*_*n*−*mM*_ to *s*_*n*+*mM*_ (see Appendix). Hence, it explains why OMA is less affected by small drifts and subject head movements than AAS.

As reported by Mandelkow et al. [25], synchronisation between the EEG sampling interval and the fMRI acquisition clock leads to increasing of the usable bandwidth in the corrected EEG up to around 150 Hz after average template subtraction. Since OMA is capable of provoking larger attenuation of the artefact activity in higher-frequency bins, this shows that our proposed approach could be used to produce further broadening of the usable bandwidth of the corrected EEG. As shown in Figures 10 and 11, by using OMA, satisfactory attenuation in the artefact frequency bins could be achieved in the bandwidth below 500 Hz. The study of high-frequency neuronal activity between 100 and 500 Hz has currently received increased attention and requires the use of customised fMRI sequences that are generally not available to all investigators [17, 20, 34]. Furthermore, it makes use of an interleaved fMRI protocol, which has been shown to be less effective and flexible than continuous fMRI measurements [9]. Therefore, the usage of OMA could be useful in EEG-fMRI studies that address those high-frequencies oscillations. As depicted in Figure 10, implementation and application of OMA also have no dependency on interpolation or slice-timing correction between the slice-length and the EEG sampling interval [14, 21, 27, 33]. This characteristic suggests that our approach can produce satisfactory results even when there are jitter errors between fMRI clock and EEG sampling rate. Albeit reduction of jitter by using hardware synchronisation solutions has become increasingly available and OMA is shown to provide better results within a scenario of alignment between TR-slice and the EEG sampling interval (Philips data), (16) reveals representing a general analytical expression that allows satisfactory estimation and suppression of the gradient artefact in scenarios either with or without the occurrence of such timing errors. Moreover, our method is data-driven, not requiring accurate information about MRI triggers and events to be implemented [14, 25, 26, 34].

The approach depicted in Figure 6 proposed for evaluation of EEG preservation is shown to simplify the implementation of this measurement, so that there is no need to perform comparison of spectral power of excerpts of the reference EEG, *e*_ref,*n*_, with corrected EEG excerpts, ${\hat{e}}_{true,n}$, as is usually performed in the literature [14, 34, 35]. Since the power spectrum represents an average measure of the frequencies contained in the time-domain signal, spectral power associated with artefact residuals might be masked in the power spectrum of ${\hat{e}}_{true,n}$, which can compromise this kind of analysis. Application of the artefact correction approach directly in the reference EEG [34, 35] also has the disadvantage of not accounting for the influence of the artefact, which might impair the accuracy of the assessment of EEG preservation by using such a procedure. Furthermore, the stochastic nature and the lack of knowledge of the neuronal EEG make it imprecise to compare the power spectrum of the artefact-corrected EEG with the spectrum of the reference EEG recorded inside or outside the scanner. Therefore, evaluation of EEG preservation by quantification of power in certain spectral bands should always be performed together with evaluation of EEG preservation in time-domain in order to obtain a more precise measurement. On the other hand, the assessment of single events is not suitable to account for the stochastic and nonperiodic nature of the neuronal EEG [35]. All these characteristics can be effectively accounted for by using the time-domain scheme depicted in Figure 6.

The selectivity of the OMA filtering, that is, the amount of attenuation of the gradient artefact together with the degree of EEG preservation, is influenced by the slice-length (TR-slice or *M*) and the values of *J* and *L*. Therefore, combination of proper values of those parameters should be taken into account to obtain an optimal balance between artefact attenuation and EEG preservation. In this respect, the assessment indicated in Tables 2 [Table tab3] [Table tab4]–5 and Figure 11 could be used to choose the values of *J* and *L* according to the value estimated for *M*, as well as obtaining an optimal selectivity. Additionally, the EEG sampling frequency should be taken into account as well, since it can influence the value of *M*. Some preliminary investigations suggested that OMA might be used to satisfactorily produce EEG correction for lower EEG sampling frequencies (around 500 Hz), provided that the gradient artefact waveforms are well reproduced in the scalp potential. Further, OMA might be applied when empty gaps occur between volume acquisitions, by setting *M* as TR-slice followed by TR, without substantial degradation of the EEG quality. These characteristics should be better assessed in future work. As further suggestion for future work, OMA should be applied and generalised for other types of EEG data recorded in other types of MR scanners as well as for correction of other types of periodic artefacts, such as the artefact that affects the scalp potential during electrical impedance tomography [50].

Because of the computational efficiency of the OMA filter (*H*_OMA_), (15) and (16) are not computationally demanding either, in addition to possessing quite low complexity of implementation. Thus, the time to compute our proposed approach is quite low and comparable with the processing time of the mean template subtraction performed by the Brain Vision Analyzer software. Equations (15) and (16) also allow using noninteger values of *M*, according to the estimated value of TR-slice, which can be used to reduce the effect of jitter errors on the comb-filtering performance as well [24]. Regarding the use of (11), some problems that arise are the substantial ringing effects in the signal ends provoked by the recursive application and subtraction of the components of the corrected EEG as well as the increased computational load. Thereby, (15) and (16) should preferably be used in order to minimise such problems. Such characteristics and the evaluation metrics utilised here constitute the innovative outcomes of this work in comparison with some previous results presented by our group [38, 41, 42]. Last, we noticed that the performance of OMA might be compromised with the occurrence of EEG amplifier saturation, as also observed for the AAS method. Thus, band limiters are needed to reduce the EEG system dynamic range and prevent saturation of the EEG amplifier during EEG data acquisition.

## 6. Conclusions

In this work, we have shown the efficacy of using optimised moving-average filtering (OMA) for suppression of the gradient artefact from the scalp potential recorded during continuous EEG-fMRI. OMA constitutes a comb-filtering approach, whose application in the scalp potential allows suppression of the gradient artefact and simultaneous estimation of the neuronal EEG. When compared with the slice-average subtraction as performed according to the established average artefact subtraction (AAS) methodology, OMA is revealed to be capable of obtaining an improved balance compared to AAS for the trade-off between effective suppression of the gradient artefact and preservation of the EEG signal. In this respect, OMA can provide larger attenuation in higher-frequency artefact bins and be less affected by artefact waveform alterations owing to small drifts and subject head motions. In addition to being data-driven and not requiring accurate information about MRI trigger and events, OMA also is shown to satisfactorily correct the EEG signal in scenarios either with or without misalignment between the EEG sampling interval and the MR slice-time. Finally, besides effectively accounting for the stochastic and nonperiodic nature of the neuronal EEG, our proposed approach for evaluation of EEG signal preservation is shown to simplify the implementation of this measurement. Such characteristics indicate that our methodology can help to improve the quality of the EEG signal recorded during fMRI as well as the performance evaluation of the gradient artefact correction approaches and thus contribute to the consolidation of coregistered EEG-fMRI.

## Figures and Tables

**Table 1:** Median artefact voltages attenuation over time.

Method	Philips data		GE data	
	RMS (*µ*V)	Amplitude (*µ*V)	RMS (*µ*V)	Amplitude (*µ*V)
OMA	622.5291	5167.6000	207.1564	1847.9000
AAS	622.5524	5172.3000	207.1422	1858.1000

**Table 2 tab2:** Median attenuation in the artefact frequency bins.

EEG data set					
Philips data			GE data		
Frequency Bin (Hz)	OMA	AAS	Frequency bin (Hz)	OMA	AAS
	Attenuation (dB)	Attenuation (dB)		Attenuation (dB)	Attenuation (dB)
20	45.3751	57.5263	14.01	16.9613	16.1357
40	78.9570	91.8088	28.01	33.6730	32.4054
60	85.6000	96.0201	42.02	60.0656	55.6886
80	98.2415	111.0143	56.02	62.9907	61.6082
100	98.3248	110.7207	70.03	47.2065	40.3737
120	110.7477	121.1444	84.03	67.6971	63.3023
140	117.0230	126.7208	98.04	70.2665	64.4877
160	104.1135	110.7799	112.04	77.2094	71.3954
180	112.6177	119.6797	126.05	76.8379	64.6367
200	120.2490	126.1687	140.06	71.6883	61.2908
220	122.7164	127.8049	154.06	92.4285	82.1534
240	120.7178	123.6904	168.07	73.6179	57.6112
260	122.9039	125.0317	182.07	103.6173	77.8464
280	116.5943	117.3843	196.08	104.8400	82.8587
300	130.2747	129.5068	210.08	111.4681	81.6893
320	141.4297	139.5853	224.09	111.8922	80.3991
340	140.0759	136.2834	238.10	127.1047	78.2103
360	142.5260	138.4374	252.10	143.5516	85.3269
380	144.0613	137.4788	266.11	149.7997	78.9997
400	140.8645	132.8269	280.11	153.8597	79.6079
420	137.6477	128.4942	294.12	174.5559	84.2324
440	142.1547	131.7775	308.12	163.7475	77.0851
460	142.6946	131.4058	322.13	173.4991	79.8261
480	141.9741	130.2354	336.13	173.0371	82.8779
500	131.2361	117.6283	350.14	179.3995	81.8668
			364.15	176.0360	81.8207
			378.15	160.9627	79.0052
			392.16	181.9554	80.8334
			406.16	163.7591	77.9195
			420.17	175.0643	79.6067
			434.17	175.9680	79.2014
			448.18	171.1805	78.3351
			462.18	171.7091	78.1379
			476.19	153.6883	77.2672
			490.20	171.5568	77.0958

**Table 3 tab3:** Median SNR and MSE considering the evaluation of the EEG preservation according to the scheme of Figure 6.

Method	Philips data		GE data	
	SNR	MSE (*µ*V^2^)	SNR	MSE (*µ*V^2^)
OMA	0.9999	0.1498	0.9993	1.1062
AAS	0.9990	2.3031	0.9960	7.1058

**Table 4 tab4:** SNR and MSE for some EEG electrodes (Philips data), considering application of low-pass filtering in *e*_ref,*n*_ and ${\hat{e}}_{ref,n}$.

Measure	Method	EEG electrode	Filter cut-off frequency					
			No LP	150 Hz	120 Hz	100 Hz	70 Hz	50 Hz
SNR	OMA	Fp1	0.9999	1.0000	1.0000	1.0000	1.0000	1.0000
		F3	0.9998	0.9999	0.9999	0.9999	0.9999	0.9999
		Oz	0.9999	0.9999	0.9999	0.9999	0.9999	0.9999
		CPz	0.9997	0.9999	0.9999	0.9999	1.0000	1.0000
		Fz	0.9997	0.9999	1.0000	1.0000	1.0000	1.0000
		FC5	0.9992	0.9998	0.9998	0.9998	0.9998	0.9998
		AF3	0.9998	0.9999	0.9999	0.9999	0.9999	0.9999

SNR	AAS	Fp1	0.9993	0.9994	0.9995	0.9995	0.9995	0.9995
		F3	0.9988	0.9990	0.9991	0.9991	0.9991	0.9992
		Oz	0.9987	0.9989	0.9989	0.9989	0.9990	0.9990
		CPz	0.9977	0.9981	0.9982	0.9983	0.9984	0.9984
		Fz	0.9987	0.9990	0.9991	0.9991	0.9991	0.9992
		FC5	0.9975	0.9986	0.9988	0.9989	0.9990	0.9990
		AF3	0.9991	0.9992	0.9993	0.9993	0.9994	0.9994

MSE (*µ*V^2^)	OMA	Fp1	0.5078	0.2047	0.1686	0.1537	0.1407	0.1359
		F3	0.5841	0.3335	0.3123	0.3020	0.2913	0.2867
		Oz	0.4108	0.1962	0.1794	0.1734	0.1694	0.1685
		CPz	0.1355	0.0283	0.0225	0.0202	0.0184	0.0179
		Fz	0.1091	0.0183	0.0148	0.0132	0.0119	0.0114
		FC5	2.3462	0.7015	0.5607	0.5026	0.4500	0.4299
		AF3	0.3380	0.1484	0.1305	0.1230	0.1161	0.1135

MSE (*µ*V^2^)	AAS	Fp1	3.6086	2.8759	2.6540	2.5278	2.3824	2.3155
		F3	3.2115	2.6447	2.4861	2.3915	2.2782	2.2246
		Oz	3.7296	3.1849	2.9991	2.8894	2.7599	2.6993
		CPz	0.9186	0.7407	0.6878	0.6575	0.6225	0.6063
		Fz	0.4204	0.3190	0.2957	0.2826	0.2675	0.2606
		FC5	7.7577	4.0962	3.4639	3.1535	2.8346	2.7007
		AF3	1.9105	1.5336	1.4264	1.3652	1.2941	1.2611

**Table 5:** SNR and MSE for some EEG electrodes (GE data), considering application of low-pass filtering in *e*_ref,*n*_ and ${\hat{e}}_{ref,n}$.

Measure	Method	EEG electrode	Filter cut-off frequency					
			No LP	150 Hz	120 Hz	100 Hz	70 Hz	50 Hz
SNR	OMA	Fp1	0.9990	0.9997	0.9998	0.9998	0.9998	0.9998
		F3	0.9974	0.9994	0.9996	0.9996	0.9997	0.9997
		Oz	0.9980	0.9992	0.9993	0.9993	0.9993	0.9994
		CPz	0.9968	0.9992	0.9994	0.9995	0.9996	0.9997
		Fz	0.9977	0.9991	0.9991	0.9991	0.9990	0.9990
		FC5	0.9958	0.9989	0.9990	0.9990	0.9990	0.9989
		AF3	0.9990	0.9997	0.9998	0.9998	0.9998	0.9998

SNR	AAS	Fp1	0.9960	0.9961	0.9962	0.9962	0.9962	0.9962
		F3	0.9977	0.9981	0.9982	0.9982	0.9982	0.9983
		Oz	0.9861	0.9861	0.9861	0.9861	0.9861	0.9861
		CPz	0.9949	0.9952	0.9953	0.9953	0.9953	0.9954
		Fz	0.9953	0.9956	0.9956	0.9957	0.9957	0.9957
		FC5	0.9974	0.9979	0.9979	0.9980	0.9980	0.9981
		AF3	0.9969	0.9971	0.9971	0.9971	0.9971	0.9971

MSE (*µ*V^2^)	OMA	Fp1	8.1852	2.2973	1.9257	1.8225	1.7843	1.7917
		F3	3.1040	0.6376	0.4770	0.4207	0.3828	0.3740
		Oz	2.5445	0.9858	0.8820	0.8359	0.7909	0.7727
		CPz	0.7211	0.1624	0.1208	0.1007	0.0807	0.0726
		Fz	0.6469	0.2537	0.2448	0.2493	0.2621	0.2703
		FC5	8.6758	2.2185	1.9761	1.9478	1.9942	2.0390
		AF3	2.3881	0.6828	0.5910	0.5671	0.5628	0.5685

MSE (*µ*V^2^)	AAS	Fp1	32.2881	30.8757	30.5258	30.3384	30.1333	30.0426
		F3	2.7148	2.1758	2.0877	2.0445	2.0002	1.9816
		Oz	18.2050	17.7786	17.6364	17.5485	17.4401	17.3874
		CPz	1.1722	1.0444	1.0174	1.0024	0.9855	0.9779
		Fz	1.3609	1.2396	1.2154	1.2021	1.1870	1.1801
		FC5	5.4794	4.1711	3.9548	3.8491	3.7422	3.6981
		AF3	7.5164	7.1415	7.0543	7.0059	6.9510	6.9259

## Appendix

Subtraction of a mean template calculated over 2*m* + 1 slice-averaging epochs could be described according to

(A.1) e^true,isi−12m+1∑k=−mmsm=etrue,i+gartf,i−12m+1∑k=−mmetrue,m+gartf,m=etrue,i+gartf,i−12m+1∑k=−mmetrue,m−12m+1∑k=−mmgartf,m,

where ${\hat{e}}_{true,i}$ is the estimate of the actual EEG in the epoch *s*_*i*_; *s*_*m*_ represents the epochs of the scalp potential considered for averaging; *e*_true,*i*_ and *g*_artf,*i*_ are the actual EEG and the gradient artefact in the epoch *s*_*i*_; and *e*_true,*m*_ and *g*_artf,*m*_ are the actual EEG and the gradient artefact in the epoch *s*_*m*_. When uncorrelation between the neuronal EEG signal and the gradient artefact is assumed, the amplitude of the averaged EEG epochs in the third term of the right hand side of (A.1) equals $1/\sqrt{2m+1}$ times the EEG RMS, in such a way that it is cancelled out by averaging. And the estimate of the average gradient artefact ${\hat{g}}_{artf,i}$ in epoch *i* corresponds to

(A.2) $$\begin{array}{c} {\hat{g}}_{artf,i}=\frac{1}{2m+1}\overset{m}{\underset{k=-m}{\sum }}g_{artf,m}. \end{array}$$

In an optimal scenario in which the gradient artefact waveform is perfectly reproducible in each epoch, the estimate ${\hat{g}}_{artf,i}$ equals *g*_artf,*i*_(= *g*_artf,*m*_), and thus ${\hat{e}}_{true,i}$ matches the actual EEG, *e*_true,*i*_. However, under realistic scanning conditions, changes in EEG recording geometry by subject motion together with inconsistent scalp potential due to systematic jitter between EEG system sampling and the fMRI clock result in alterations in the artefact waveform. This generates a component of uncertainty in the estimate ${\hat{g}}_{artf,i}$ that must be inserted in (A.2):

(A.3) g^artf,i=12m+1∑k=−mmgartf,m+um=gartf,i+12m+1∑k=−mmum.

Thus, ${\hat{e}}_{true,i}$ in (A.1) could be expressed as the sum of the actual EEG, *e*_true,*i*_, with the uncertainty *u*_*i*_:

(A.4) $$\begin{array}{c} {\hat{e}}_{true,i}=e_{true,i}+\frac{1}{2m+1}\overset{m}{\underset{k=-m}{\sum }}u_{m}=e_{true,i}+u_{i}. \end{array}$$

Therefore, the residual artefact that remains in the corrected EEG after AAS is associated with a component of uncertainty that represents the average of the particular uncertainties of samples belonging to the 2*m* + 1 averaging epochs taken into account for calculation of the artefact template. Hence, the influence of small drifts and subject head movements that occur in scalp potential samples of 2*m* + 1 epochs (ranging from *s*_*n*−*mM*_ to *s*_*n*+*mM*_) has an impact over the uncertainty *u*_*i*_ [15].

On the other hand, as described in (2), the artefact is estimated sample-by-sample through the moving-averaging filter when the optimised moving-average (OMA) is applied:

(A.5) 1M∑k=0M−1sn−k1M∑k=0M−1etrue,n−k+gartf,n−k=1M∑k=0M−1etrue,n−k+1M∑k=0M−1gartf,n−k=e^n¯+1M∑k=0M−1gartf,n−k.

When an optimal scenario is assumed, the gradient artefact waveform is exactly stationary in the period TR-slice (= *M*), and the term corresponding to the artefact in (A.5) is cancelled out by integration, as indicated in (3). Under realistic conditions, however, the influence of subject head motions and jitter generates a component of uncertainty in (A.5):

(A.6) 1M∑k=0M−1sn−k=e^n¯+1M∑k=0M−1gartf,n−k+un−k,1M∑k=0M−1sn−k=e^n¯+1M∑k=0M−1gartf,n−k+1M∑k=0M−1un−k.

Thereby, since the uncertainty component has been taken into consideration in (A.6), the term corresponding to the gradient artefact can precisely be cancelled out by integration. Backward application of the moving-average in (A.6) results in

(A.7) 1M∑k=M−101M∑k=0M−1sn−kn+k=1M∑k=M−10e^¯+1M∑k=0M−1un−kn+k=ecomp,1,n+1M2∑k=−M+1M−1M−kun+k=ecomp,1,n+ucomp,j,n.

And, thus, the uncertainty *u*_comp,*j*,*n*_ corresponds to

(A.8) ucomp,j,n=1M2un−M+1+2un−M+2+⋯+M−1un−1+Mun+M−1un+1+⋯+M−1un+1+2un+M−2+un+M−1.

Therefore, in case of the OMA filter, the uncertainty is influenced by jitter and subject head movements that occur in the samples of the scalp potential raging from *s*_*n*−*M*+1_ to *s*_*n*+*M*−1_. This characteristic allows reducing the impact of small drifts and head movements by using OMA in comparison with the AAS method, since the uncertainty associated with the corrected EEG by OMA is influenced by samples located in a shorter length of signal than the EEG corrected by AAS averaging.
